# Using Peer Crowd Affiliation to Address Dual Use of Cigarettes and E-Cigarettes among San Francisco Bay Area Young Adults: A Cross Sectional Study

**DOI:** 10.3390/ijerph17207643

**Published:** 2020-10-20

**Authors:** Nhung Nguyen, Louisa M. Holmes, Minji Kim, Pamela M. Ling

**Affiliations:** 1 Center for Tobacco Control Research and Education, University of California San Francisco, San Francisco, CA 94143, USA; minji.kim@ucsf.edu (M.K.); Pamela.Ling@ucsf.edu (P.M.L.); 2 Departments of Geography & Demography, and the Social Science Research Institute, Penn State University, University Park, PA 16802, USA; lmholmes@psu.edu

**Keywords:** tobacco, dual tobacco use, psychographics, emerging adults, electronic cigarettes, vaping, smoking

## Abstract

Given the emerging tobacco landscape, dual use of cigarettes and e-cigarettes has increased among young adults, but little is known about its associated factors. Peer crowds, defined as macro-level connections between individuals with similar core values (e.g., “Hip Hop” describing a group that prefers hip hop music and values strength, honor, and respect), are a promising way to understand tobacco use patterns. We examined associations between peer crowds and tobacco use patterns by using data from a cross sectional survey of 1340 young adults in the San Francisco Bay Area in 2014. Outcomes were the past 30-day use of: neither cigarettes nor e-cigarettes; cigarettes but not e-cigarettes; e-cigarettes but not cigarettes; and both cigarettes and e-cigarettes. Peer crowds included Hipster, Hip Hop, Country, Partier, Homebody, and Young Professional. Multinomial regression analysis indicated that peer crowds were significantly associated with different tobacco use patterns. Compared to Young Professionals, Hip Hop and Hipster crowds were more likely to dual use; Hipsters were more likely to use e-cigarettes only, and Country participants were more likely to smoke cigarettes only. These findings suggest that tobacco control campaigns and cessation interventions should be tailored to different young adult peer crowds and address poly-tobacco use.

## 1. Introduction

Tobacco use among young adults (aged 18–26) is a major public health concern as young adulthood includes the peak time of progression from experimentation to regular tobacco use [1]. The tobacco landscape has shifted from conventional cigarettes to include cigars/cigarillos, hookah, smokeless tobacco, and more recently electronic cigarettes (e-cigarettes) [2]. This changing product landscape has resulted in a transformation of tobacco use patterns with the use of e-cigarettes now surpassing conventional cigarette smoking among adolescents and increasing rates of concurrent use of two or more tobacco products (poly-tobacco use) [3,4,5]. According to national data among US adults in 2018, 19.7% currently used any tobacco product and 3.7% used multiple tobacco products [6]. While cigarette smoking reached an all-time low (13.7%), prevalence of e-cigarette use increased from 2.8% to 3.2% during 2017–2018. Compared to other adult age groups, young adults had the lowest prevalence of cigarette use (7.8%) but the highest prevalence of e-cigarette use [6] and of poly-tobacco use (22%), and the most common combination was dual use of cigarettes and e-cigarettes [7]. Notably, dual use of cigarettes and e-cigarettes is increasing among US young adults, accompanying escalating use of e-cigarettes [1,4,5,8]. This dual use pattern may place young adults at risk of higher nicotine exposure, increased negative health effects, and decreased tobacco cessation [9,10]. National data indicated that dual use in the general population was more prevalent among males (vs. females), White (vs. Hispanic or Black) adults, and those with less than high school education [1]. However, very little information exists on factors related to dual use among young adults beyond demographic factors. Understanding characteristics of dual users, and to what extent dual users differ from single users (e.g., cigarette or e-cigarette only users) and non-users is needed to inform tobacco prevention and intervention efforts. 

As smoking rates continue to decline, tobacco companies are moving into the vaping industry and using social media to promote their products. Recent research indicated that social media platforms (e.g., Instagram, YouTube) are dominated by pro-vaping messages disseminated by the tobacco industry and that exposure to e-cigarette online marketing is associated with lower harm perceptions of e-cigarettes, greater intention to use e-cigarettes, and increase in e-cigarette trial in adolescents and young adults [11,12]. In addition, the tobacco industry has historically used psychographic approaches to segment and target young adults in order to market their products based on lifestyles [13], and this has been shown to increase young tobacco users’ engagement [14]. For example, tobacco companies have targeted young people of color using Hip Hop culture and music [15,16]. Mirroring this, a recent development in tobacco control programs is to tailor programs using peer crowd affiliation [17,18]. Peer crowds are defined as macro-level connections between individuals with similar core values, interests, and lifestyles across geographic areas [19]. Common peer crowds among US young adults include “Hipster” (value creativity and individuality, prefer indie rock music, and set trends), “Hip Hop” (prefer hip hop music and value strength, honor, and respect), “Country” (prefer country music, engage in outdoor activities, and value tradition, hard work, patriotism, community, and family), “Partier” (prioritize success and go out to bars and nightclubs), “Homebody” or “Mainstream” (prefer a quite night at home and prioritize family, career, and religion), and “Young Professionals” (value their careers and prioritize networking) [20,21,22,23,24]. 

Emerging evidence showed that peer crowd affiliation has been associated with a variety of health behaviors among adolescents and young adults, including substance use, depression, bullying, violence, and obesity [19,21,25,26]. In the context of tobacco use, peer crowd affiliation consistently predicted tobacco use among young adults independent of demographic characteristics. For example, Young Professional and Homebody groups demonstrated lower risks of tobacco use, while Hip Hop demonstrated a higher risk of tobacco use [20,21,27]. Given its association with tobacco use behaviors, peer crowd affiliation can be a promising criterion for audience segmentation in targeted communications to reduce tobacco use [18,28]. For example, national targeted media campaigns, such as the Food and Drug Administration (FDA)’s “Fresh Empire” tobacco prevention campaign, have been shown to effectively reach Hip Hop adolescents [17,29]. Recent research also suggests that peer crowd targeting significantly increased the efficiency with which anti-tobacco interventions could reach at-risk young adult smokers [27,30,31]. One study among 3368 young adults found that the Hip Hop and Hipster peer crowds reported the most frequent use of cigarettes, e-cigarettes, and cigars [20]. Another study used a nationally representative sample of 1341 young adults and found that the Partier peer crowd was more likely to use cigarettes and e-cigarettes while the Country crowd was more likely to use cigarettes and smokeless tobacco [21]. However, extant research on peer crowd affiliation is still limited in understanding poly-tobacco use among young adults, particularly dual use of cigarettes and e-cigarettes, which has become the most popular tobacco use pattern for this age group [1,4,5,8]. 

To address the aforementioned gaps, using data from a representative sample of young adults in the San Francisco Bay Area, this study aimed to examine the association between peer crowd affiliation and mutually exclusive patterns of e-cigarette and cigarette use (i.e., neither use of cigarettes nor e-cigarettes; use of cigarettes but not e-cigarettes; use of e-cigarettes but not cigarettes; and dual use). We hypothesized that peer crowd affiliation would be associated with these patterns independent of demographic characteristics. By addressing peer crowds at risk of dual tobacco use, this study may inform public health campaigns in order to tailor efforts to prevent tobacco use among this population more effectively.

## 2. Materials and Methods

### 2.1. Study Design 

This study used data from the 2014 San Francisco Bay Area Young Adult Health Survey, a probabilistic household study of young adults in Alameda and San Francisco Counties in California. Methods have been described in detail elsewhere [27]. Briefly, we utilized a multimode multistage stratified probability sampling method to collect data in a manner that ensured appropriate representation by geography, age, sex, and race/ethnicity. We identified potential households using address lists obtained from the Marketing Systems Group with approximately 40% chance that a young adult resided at a selected address (*n* = 15,000 addresses). We also used the 2009–2013 American Community Survey and 2010 decennial census data in a multistage sampling procedure to supplement the address-based sample. We identified Census Blocks in which at least 15% of residents were in the eligible age range (*n* = 1636 housing units) in order to randomly select blocks with higher concentrations of Black and Latino young adults (*n* = 61), as these populations are particularly hard to reach. We employed four modes of survey delivery (mail, web, telephone, face-to-face). Since the participants were young adults (≥ 18 years old), there was no need to obtain consent to participate from the parents or legal guardians. Informed consent was obtained from all participants. The study was approved by the IRB at the University of California, San Francisco (#13-11907). The dataset generated for the current study are available in the Figshare repository (https://figshare.com/s/1995f791d257dac75500)

### 2.2. Study Participants

The final sample consisted of 1340 young adults, reflecting a response rate of approximately 30%. Survey completion rates varied by mode. Approximately two-thirds of completed questionnaires were returned via mail or completed online. Most of the remaining responses were obtained through household visits. Only three participants completed the questionnaire via telephone, and this mode was primarily used to eliminate ineligible households from the sample.

Acceptable survey response rates range widely [32] and tend to be lower among hard-to-reach populations, such as urban young adults, the population most difficult to enroll in surveys [33]. As such, many studies of young adults rely on convenience samples (e.g., college student populations) which may have higher response rates but do not represent the population. It is difficult to gauge our survey response rate against similar surveys because we are unaware of any similar probabilistic regional household surveys of young adults to which we can compare rates. However, national surveys of adolescents, such as the Youth Risk Behavior Survey and AddHealth, have demonstrated response rates ranging from 23% to 91% depending on mode of survey delivery [34,35]. An amplified effort to increase response rates among young adults to the National Survey on Drug Use and Health “boasted” a 30–40% response rate [36]. To our knowledge, our study was the first probabilistic multimode household study of young adults in this age range. Despite the low response rate, our sample was representative of the population we sought to survey. We compared the sample distribution to American Community Survey public use microdata sample estimates to verify that our sample distribution closely matched the distribution of the 18–26-year-old population in the two counties by location, age, race/ethnicity, and sex. We also created post-stratification weights for the sample to ensure representation according to these characteristics. 

### 2.3. Measures

#### 2.3.1. Tobacco Use 

Adopted from US national surveys on substance use among youth and young adults [3,4,18,37,38], current tobacco use was assessed by an item “During the past 30 days, on how many days did you use/smoke at least…?”, followed by a list of tobacco products (one cigarette, an e-cigarette, one cigar/cigarillo, a hookah, and smokeless tobacco (spit tobacco, chew, moist snuff, snus)). Current use for each product was defined as use of that product on at least 1 day in the past 30 days. For our analyses, the current use of any of cigars/cigarillos, hookah, and smokeless tobacco products were combined into “other tobacco use”, and this variable was used as a covariate. 

#### 2.3.2. Outcomes 

The outcomes were derived from two measures (i.e., “current cigarette use” and “current e-cigarette use”), and categorized into four mutually exclusive categories: 0 = “not currently using either cigarettes or e-cigarettes”; 1 = “currently using cigarettes but not e-cigarettes”; 2 = “currently using e-cigarettes but not cigarettes”; and 3 = “currently using both cigarettes and e-cigarettes” [4,39]. 

#### 2.3.3. Independent Variable 

Peer crowd affiliation was measured using the I-Base Survey^®^ [20,24]. Extensive qualitative research on young adults’ lifestyles and values that determined the macro-level peer crowds was conducted as part of the formative research for several young adult tobacco interventions [31,40,41,42]. Based on our previous work, we identified six peer crowds (i.e., Hip Hop, Hipster, Country, Partier, Homebody, and Young Professional) commonly found among young adults in different regions of the US, and employed the I-Base Survey^®^ to measure young adults’ affiliation with these peer crowds. This instrument has been used widely and demonstrated effectiveness and consistency in identifying increased health risk behaviors among adolescent and young adult peer crowds in California and other states [18,20,25,43]. 

The I-Base Survey^®^ is a picture-based survey instrument designed to measure peer crowd affiliation. It is a proprietary tool created by Rescue Agency and has been used in this study under license. This tool included a grid of photos of young adults of diverse race/ethnicity and gender (36 males, 36 females) with each photo pre-assigned to one of the 6 exclusive peer crowds. Examples of the photos are presented elsewhere [14]. Participants were asked to choose three male and three female photos that best fit their main group of friends and another three that least fit. The score for each peer crowd were summed based on ranks of photo selection (i.e., 3, 2, 1 for “the best fit”; and −3, −2, −1 for “the least fit”) with a total score of a range from −12 to 12. Participants were classified to a certain peer crowd based on their highest scores. For example, if a person scored 8 points on Hipster photo selection and 4 points on Partier photo selection, they would be classified as Hipster. Participants with tied scores (*n* = 121) were randomly assigned to one of the highest-scored peer crowds. For the participants with tied peer crowd scores, random numbers were generated in Stata v14 using the “rand” command. For ties between two peer crowds, each peer crowd was also randomly assigned a value of “1” or “0;” respondents with a random value below 0.5 were assigned to the peer crowd valued “0,” and those with a random value equal to or greater than 0.5 were assigned to the peer crowd valued “1.” For ties between three peer crowds, values between 1–3 were randomly assigned to the peer crowds, and respondents were assigned based on tertiles: >0 and ≤0.33; >0.33 and ≤0.66; and >0.66 and ≤1. More details about peer crowd scoring have been provided elsewhere [14,27]. 

#### 2.3.4. Other Covariates

Demographic characteristics (i.e., age, sex, race/ethnicity, educational attainment) were obtained. Age was calculated based on self-reported date of birth. Sex at birth was dichotomized as female and male. Race/ethnicity was categorized into Hispanic, non-Hispanic White, Black, Asian/Pacific Islander, and Multiracial. Educational attainment was dichotomized as “Less than college” and “Currently enrolled in college or had a bachelor degree or higher”, since having some college education was a documented predictor of tobacco use among young adults [44]. Current use of alcohol was defined as using at least one drink of alcohol (bottle of beer, shot of liquor, glass of wine) on at least one day during the past 30 days, based on measures of alcohol use in other national surveys on drug use among young populations [37]. 

### 2.4. Statistical Analysis

All analyses were weighted using person-level weights to adjust for the complex sampling design and clustering. Descriptive statistics were computed from the complete data for the total sample and for each outcome category. Current use of tobacco products (i.e., cigarette, e-cigarette, and other tobacco) were described for each peer crowd. Missing data were addressed using multiple imputation. We assumed that tobacco use and peer crowd variables were missing at random, conditional on prior observed responses. We imputed all variables (i.e., the independent variable, covariates, and the outcome) in the analysis model and other auxiliary variables (i.e., “smoked more than 100 cigarettes lifetime” and “ever smoked daily”) [45]. We then applied multiple imputation via chained equations to create imputed data sets. Multinomial logistic regression models on the imputed data examined the association between peer crowd affiliation and outcomes, adjusting for demographic variables (i.e., age, sex, race/ethnicity, educational attainment), other tobacco use, and alcohol use. These covariates were selected based on previous studies [27,46]. The Young Professional peer crowd was used as the reference group in order to be consistent with our previous research [27]. All tests of hypotheses were two-tailed with a significance level of α less than 0.05. Data were analyzed using “mi” and “svy” commands in STATA 15 (StataCorp LLC, College Station, TX, USA).

## 3. Results

### 3.1. Sample Characteristics

The study sample characteristics are shown in Table 1. Participants were on average 22.7 years old (SD = 2.5) with a range of 18–26 years old. The sample was half female (50.4%), and racially/ethnically diverse with 31.9% non-Hispanic White. About two thirds of dual users were Hispanic (36.4%) and non-Hispanic White (36.0%). Participants had relatively high levels of education with 83.1% either currently enrolled in college or having a bachelor’s degree or higher. Regarding peer crowd affiliation, more young adults identified with the Young Professional (37.1%) and Homebody (34.3%) than with Hipster (7.1%), Hip Hop (8.4%), Partier (8.8%), or Country (4.4%) in our sample. Overall, 20.2% of the sample were current users of e-cigarettes and/or cigarettes, and 17.2% reported any current use of other tobacco products. Of note, there were roughly equal sized groups of cigarette only users (7.0%), e-cigarette only users (6.5%), and dual users (6.7%). 

### 3.2. Tobacco Use by Peer Crowd

Tobacco use measures for each peer crowd are displayed in Table 2. Patterns of e-cigarette and cigarette use differed significantly across the six peer crowds. Overall estimates of current use of any cigarettes and/or e-cigarettes ranged from 11.6% among Young Professionals to 45.8% among Hip Hop respondents. Regarding specific patterns, e-cigarette only use was the most common pattern for Hipster (14.5%), Partier (14.3%), and Young Professional (5.6%) respondents, while cigarette only use was most frequent among Hip Hop (24.2%) and Country (25.3%), and dual use for Homebody (7.2%). Proportions of dual use were highest for Hip Hop (15.0%), followed by Hipster (14.3%), Partier (12.3%), Country (9.9%), Homebody (7.2%), and Young Professional (2.1%). In terms of specific products, e-cigarettes were reported most for Hipster (28.8%) and Partier (26.6%) peer crowds, while cigarettes were the most popular product used among Hip Hop (39.2%), Country (35.2%), and Homebody (12.9%) participants. Poly-tobacco use was higher than single use for Hip Hop and Hipster, but not for the other peer crowds.

### 3.3. Associations between Peer Crowd Affiliation and Patterns of Tobacco Use

Adjusting for other covariates, the multinomial logistic regression models on the imputed data indicated that peer crowd affiliations were significantly associated with different patterns of e-cigarette and cigarette use (Table 3). Compared to Young Professionals, Hip Hop young adults were more likely to smoke cigarettes only (Adjusted Odds Ratio (AOR) = 9.7, 95% Confidence Interval (95%CI) = 2.9–33.1) or to dual use cigarettes with e-cigarettes (AOR = 8.7, 95%CI = 2.0–38.4). Likewise, Hipsters were more likely to use e-cigarettes only (AOR = 3.6, 95%CI = 1.3–10.6) or to dual use (AOR = 8.3, 95%CI = 2.1–33.5). Country peer crowd members were more likely to smoke cigarettes only (AOR = 8.4, 95%CI = 2.6–26.6). Current use of other tobacco products and alcohol were positively associated with all the patterns of e-cigarette and cigarette use. 

## 4. Discussion

This study is among the first to provide evidence on the association between peer crowd affiliation and four mutually exclusive patterns of e-cigarette and cigarette use (not currently using either cigarettes or e-cigarettes; currently using cigarettes but not e-cigarettes; currently using e-cigarettes but not cigarettes; and currently using both cigarettes and e-cigarettes) among US young adults. As hypothesized, we found differential patterns of and significant associations with e-cigarette and cigarette use among the six young adult peer crowds, highlighting the risk of dual tobacco use among Hipster and Hip Hop young adults and the risk of cigarette smoking among Country young adults.

The main finding was that the Hipster and Hip Hop peer crowds were at higher risks of dual use, with more than eight times the odds of using both cigarettes and e-cigarettes compared to Young Professionals. Indeed, our estimates of dual use ranged from 2% among Young Professionals to 14–15% among Hipster and Hip Hop peer crowds. This finding is somewhat consistent with prior studies, which found that peer crowds exhibited differential risks of tobacco use [20,21,27,47]. Our study extends the peer crowd literature by identifying high-risk peer crowds of cigarette and e-cigarette dual use. This finding suggests that future research should take peer crowd affiliation into account in addressing dual tobacco use as the Hipster or Hip Hop peer crowds may increase risk for using both products. 

In addition, while our study focused on dual use, we found peer crowd affiliation predicting other tobacco use patterns. Previous studies often discussed the Country peer crowd’s higher odds of smokeless tobacco use [23,48,49,50], but our study and a recent study [21] indicated that this group also had higher odds of cigarette smoking compared to Young Professionals. This finding suggests that preventing cigarette smoking should be added to tobacco prevention targeting Country young adults. Moreover, we also found that other tobacco product use was common and positively associated with the use of cigarettes and/or e-cigarettes consistently across all the peer crowds. This finding adds to existing evidence identifying poly-tobacco use as an emerging public health problem among young people. Compared to national estimates [1,4,5,8], our study revealed even higher estimates of poly-tobacco use among the Hip Hop (34%), Hipster (24%), and Partier (23%) peer crowds. Such high prevalence of poly-tobacco use is noteworthy as our study was conducted in the San Francisco Bay Area, where has very strong tobacco control policies. This finding calls for more comprehensive educational campaigns addressing all types of tobacco or nicotine use. 

There are several potential explanations for increased risk of tobacco use among certain young adult peer crowds. As the youngest legal population for tobacco marketing, young adults are a priority target of tobacco industry marketing [51]. Increase in young adult exposure to e-cigarette advertisements [52] is concerning as exposure to tobacco advertising is an important contributor to tobacco use among young people [53,54]. E-cigarette advertising receptivity (without exposure to cigarette advertising) was associated with subsequent dual use among never tobacco users [55], suggesting the role of tobacco marketing on dual use. Moreover, tobacco companies have historically used psychographic segmentation analogous to peer crowd targeting to promote their products [15,16,53]. A recent study documented that nearly half of leading Hip Hop music videos contained combustible or electronic tobacco products, suggesting this media source may contribute to normative perceptions of tobacco use in Hip Hop culture [56]. Tobacco companies have also targeted Hipsters and other trendsetters, who adopt new products and behaviors before their peers [40,53,57]. Likewise, research indicated that the tobacco industry implemented smokeless tobacco marketing campaigns targeting Country youth and young adults [48,49,50]. Furthermore, since each peer crowd has a unique set of values and norms, their motivation to use tobacco products may be different [19,20,21]. A recent qualitative study among California young adults reported that e-cigarettes were positioned as a marker of Hipster culture to produce a “very cool and trendy” look [47]. Indeed, we found e-cigarettes were the most popular tobacco product in the Hipster peer crowd, suggesting that promotion to Hipster early adopters might contribute to the increased rates of e-cigarette use and dual use among this peer crowd. Of note, San Francisco Bay Area is the location of the JUUL company headquarters, which may also contribute to the popularity of e-cigarette use in our sample, particularly among Hipsters. 

Collectively, our findings have practical implications for reducing tobacco use, specifically dual use of cigarettes and e-cigarettes, among young people. From a public health perspective, a peer crowd-based targeting approach may be promising to reach priority young adult groups for tobacco control campaigns. Given the variability of peer crowd affiliation and its association with tobacco use patterns among young adults, a generic intervention would be limited in its ability to meaningfully address high-risk subgroups [58]. In a worst-case scenario, generic messaging may increase tobacco use disparities by leaving higher risk segments behind. Thus, an effectively targeted intervention to reduce tobacco use needs to identify high-risk target audiences (e.g., Hip Hop, Hipster, and Country peer crowds) and reflect their unique characteristics to enhance perceived relevance of public health campaigns [59,60]. To date, there have been several peer crowd targeted campaigns, including the FDA’s “Fresh Empire” campaign for Hip Hop youth [24], the “Down and Dirty” campaign for Country youth [23], the “Commune” campaign for Hipster young adult bar patrons [40], and the “HAVOC” campaign for Partier young adults [31,41]. However, these campaigns have focused on single product use (combustible cigarettes or smokeless tobacco) and we are not aware of peer crowd targeted campaigns addressing dual use of e-cigarettes and cigarettes among young adults. Our study suggests that campaigns targeting Hip Hop and Hipster peer crowds should address dual use of e-cigarettes and cigarettes rather than solely cigarette smoking in order to curb use of both products. Likewise, campaigns targeting Country young adults should take into account cigarette smoking in addition to smokeless tobacco use. In addition, although the Homebodies and Young Professionals had lower risk of tobacco use, due to their sizes in the general young adult population, these groups accounted for large numbers of tobacco users (e.g., 35% dual users were Homebodies and 31% e-cigarette only users were Young Professionals). Thus, tailored interventions addressing tobacco use among these low risk groups are also needed to reduce absolute numbers of young tobacco users. 

From a clinical perspective, quitting smoking before age 30 significantly reduces tobacco related morbidity and mortality [61]; however, young adults are less likely to receive smoking cessation assistance [40]. This study suggests that healthcare providers should screen for all types of tobacco/nicotine use in addition to smoking, particularly when treating young adults. Understanding the different motivations of different peer crowds may also facilitate more effective clinical counseling for cessation. Finally, poly-tobacco users have greater nicotine dependence [62] and lower quitting intention [63] compared to single tobacco product users. Thus, cessation interventions for dual and poly-tobacco users may need stronger efforts to identify and treat nicotine dependence, with higher priority for young adults who affiliate with high risk peer crowds. 

Our study is subject to several limitations. Due to the cross-sectional design, we cannot establish causal relationships between peer crowd affiliation and tobacco use. While we controlled for multiple demographic factors and risk behaviors, the analyses did not control for sensation seeking or other personality characteristics. The self-reported data might be subject to recall and social desirability bias. In addition, our data are restricted to young adults in the San Francisco Bay Area and the findings might not be generalizable to other locations. More research is warranted to confirm our findings of the role of peer crowd affiliation on tobacco use behaviors among young adults in other regions. Finally, since our survey was conducted in 2014, the results may not reflect current patterns of tobacco use among young adults. Despite these limitations, given the salience of dual use of e-cigarettes and cigarettes among young adults and limited research on this public health issue, the current study contributes important insights and implications to address this important pattern of tobacco use behaviors among young populations.

## 5. Conclusions

This study provides the first evidence on the relationship between peer crowd affiliation and dual use of cigarettes and e-cigarettes among San Francisco Bay Area young adults. Since tobacco use patterns differed by young adult peer crowd, tobacco control interventions should be tailored to peer crowd affiliation. Interventions focusing on dual use of cigarettes and e-cigarettes may be an effective way to prevent and reduce tobacco use among Hip Hop and Hipster peer crowds, and interventions focusing on cigarette smoking may be relevant for the Country peer crowd. In addition, given poly-tobacco use is an emerging public health problem, tobacco control campaigns and cessation interventions should address the use of all types of tobacco or nicotine among young adults.

## Figures and Tables

**Table 1:** Sample characteristics (weighted %).

Characteristics	Total	Neither Use of Cigarettes Nor E-Cigarettes	Use of Cigarettes but Not E-Cigarettes	Use of E-Cigarettes but Not Cigarettes	Dual Use of Cigarettes and E-Cigarettes	*p*-Value
Observations (Weighted %)	1340 (100.0)	1079 (79.8)	92 (7.0)	90 (6.5)	79 (6.7)	
Peer crowd affiliation						<0.01
Hip Hop	8.4	5.8	25.9	8.0	17.8	
Hipster	7.1	5.7	7.4	15.3	14.4	
Country	4.4	3.3	14.1	4.0	6.1	
Partier	8.8	7.3	9.4	18.8	15.4	
Homebody	34.3	36.1	24.9	23.1	35.1	
Young Professional	37.1	41.8	18.4	30.9	11.2	
Demographics						
Age, mean (SD)	22.7 (2.5)	22.7 (2.5)	22.6 (2.5)	22.5 (2.6)	22.5 (2.3)	0.39
Gender (Male)	49.6	47.6	56.7	56.4	59.7	0.25
Race/ethnicity						0.02
Hispanic	23.8	21.9	18.7	41.0	36.4	
NH White	31.9	31.0	43.5	25.9	36.0	
NH Black	10.1	10.0	14.7	3.6	12.1	
NH API	27.7	30.7	15.6	19.2	13.6	
NH Other	6.5	6.5	7.5	10.4	2.0	
Education						<0.01
<College	16.8	14.4	23.9	16.6	39.3	
≥College	83.1	85.6	76.1	83.4	60.7	
Tobacco use						
Ever smoked ≥100 cigarettes	13.2	4.9	53.9	19.5	64.1	<0.01
Ever smoked daily	10.2	3.7	40.3	16.3	49.5	<0.01
Past 30-day use of other tobacco	17.2	9.9	37.9	54.7	46.9	<0.01
Other						
Past 30-day use of alcohol	68.8	64.4	82.9	92.6	84.9	<0.01

Note: the weighted percentages sum up to 100% within each column; NH = Non-Hispanic; API = Asian/Pacific Islander; p-values were for ANOVA or χ^2^ tests.

**Table 2:** Tobacco use characteristics by peer crowd (weighted %).

Tobacco Use Characteristics	Hip Hop *n* = 74 (100%)	Hipster *n* = 80 (100%)	Country *n* = 61 (100%)	Partier *n* = 89 (100%)	Homebody *n* = 414 (100%)	Young Professional *n* = 440 (100%)	*p*-Value for Overall χ^2^ Tests
Patterns of current tobacco use (outcome)							<0.01
Neither use of cigarettes nor e-cigarettes	54.4	63.0	58.7	65.1	82.6	88.4	
Use of cigarettes but not e-cigarettes	24.2	8.2	25.3	8.3	5.7	3.9	
Use of e-cigarettes but not cigarettes	6.4	14.5	6.1	14.3	4.5	5.6	
Dual use of both cigarettes and e-cigarettes	15.0	14.3	9.9	12.3	7.2	2.1	
Current use of specific product							
Cigarettes	39.2	22.5	35.2	20.6	12.9	6.0	<0.01
E-cigarettes	21.3	28.8	15.9	26.6	11.7	7.7	<0.01
Hookah	16.0	12.5	2.0	22.8	11.1	8.4	0.04
Cigars/Cigarillos	18.2	11.1	8.3	12.7	7.6	3.5	0.03
Smokeless tobacco	2.0	0.5	5.9	3.8	2.9	1.1	0.55
Single use (only 1 product)	21.0	13.3	25.8	23.7	13.6	9.5	<0.01
Poly-tobacco use (≥2 products)	34.0	24.4	19.8	22.9	12.6	7.4	
Cigarette smoking history							
Ever smoked ≥100 cigarettes	28.9	19.1	23.2	31.3	10.4	7.1	<0.01
Ever smoked daily	21.5	11.6	19.8	19.0	9.1	6.1	<0.01

**Table 3:** Differential associations between peer crowd affiliation and patterns of current cigarette and e-cigarette use.

Patterns of Current Tobacco Use (Ref = Neither Use of Cigarettes Nor E-Cigarettes)	Use of Cigarettes But Not E-Cigarettes AOR (95%CI)	Use of E-Cigarettes But Not Cigarettes AOR (95%CI)	Dual Use of Both Cigarettes and E-Cigarettes AOR (95%CI)
Peer crowd			
Hip Hop	9.7 (2.9–33.1) ***	1.4 (0.3–6.0)	8.7 (2.0–38.4) **
Hipster	3.0 (0.8–10.7)	3.6 (1.3–10.6) *	8.3 (2.1–33.5) **
Country	8.4 (2.6–26.6) ***	1.7 (0.3–9.6)	4.4 (0.8–23.9)
Partier	2.0 (0.4–9.2)	1.8 (0.6–5.7)	3.6 (0.9–14.7)
Homebody	1.5 (0.6–4.0)	0.8 (0.3–2.1)	2.8 (0.9–8.2)
Young Professional	Ref	Ref	Ref
Covariates			
Current use of other tobacco	4.1 (1.8–9.3) **	9.0 (4.7–17.6) ***	6.0 (2.8–12.6) ***
Current use of alcohol	2.8 (1.2–6.8) *	5.1 (1.7–15.8) **	3.4 (1.3–9.2) *

Note: *: *p* < 0.05, **: *p* < 0.01, ***: *p* < 0.001.; AOR: Adjusted Odds ratio. CI: Confidence Interval.; The multivariate model was controlled for demographics variables (age, gender, race/ethnicity, education).

## References

[1] U.S. Department of Health and Human Services (2016). E-Cigarette Use Among Youth and Young Adults: A Report of the Surgeon General—Executive Summary.

[2] O’Connor R.J. (2012). Non-cigarette tobacco products: What have we learnt and where are we headed?. Tob. Control..

[3] Wang T.W., Gentzke A., Sharapova S., Cullen K.A., Ambrose B.K., Jamal A. (2018). Tobacco Product Use Among Middle and High School Students—United States, 2011–2017. MMWR Morb. Mortal. Wkly. Rep..

[4] Johnson A.L., Collins L.K., Villanti A.C., Pearson J.L., Niaura R.S. (2018). Patterns of Nicotine and Tobacco Product Use in Youth and Young Adults in the United States, 2011–2015. Nicotine Tob. Res..

[5] Kasza K.A., Ambrose B.K., Conway K.P., Borek N., Taylor K., Goniewicz M.L., Cummings K.M., Sharma E., Pearson J.L., Green V.R. (2017). Tobacco-Product Use by Adults and Youths in the United States in 2013 and 2014. N. Engl. J. Med..

[6] Creamer M.R., Wang T.W., Babb S., Cullen K.A., Day H., Willis G., Jamal A., Neff L. (2019). Tobacco Product Use and Cessation Indicators Among Adults—United States, 2018. MMWR Morb. Mortal. Wkly. Rep..

[7] Osibogun O., Taleb Z.B., Bahelah R., Salloum R.G., Maziak W. (2018). Correlates of poly-tobacco use among youth and young adults: Findings from the Population Assessment of Tobacco and Health study, 2013–2014. Drug Alcohol Depend..

[8] Maglia M., Caponnetto P., Di Piazza J., La Torre D., Polosa R. (2018). Dual use of electronic cigarettes and classic cigarettes: A systematic review. Addict. Res. Theory.

[9] Kalkhoran S., Glantz S.A. (2016). E-cigarettes and smoking cessation in real-world and clinical settings: A systematic review and meta-analysis. Lancet Respir. Med..

[10] West J.C., Villanti A.C., Graham A.L., Mays D., Mermelstein R.J., Higgins S.T. (2019). Tobacco Use and Cessation Behaviors in Young Adults: 2016 National Health Interview Survey. Am. J. Public Health.

[11] Collins L., Glasser A.M., Abudayyeh H., Pearson J.L., Villanti A.C. (2019). E-Cigarette Marketing and Communication: How E-Cigarette Companies Market E-Cigarettes and the Public Engages with E-cigarette Information. Nicotine Tob. Res..

[12] Tan A.S.L., Bigman C.A. (2020). Misinformation About Commercial Tobacco Products on Social Media-Implications and Research Opportunities for Reducing Tobacco-Related Health Disparities. Am. J. Public Health.

[13] Ling P.M., Glantz S.A. (2002). Using tobacco-industry marketing research to design more effective tobacco-control campaigns. JAMA.

[14] Kim M., Olson S., Jordan J.W., Ling P.M. (2020). Peer crowd-based targeting in E-cigarette advertisements: A qualitative study to inform counter-marketing. BMC Public Health.

[15] Cruz T.B., Wright L.T., Crawford G. (2010). The menthol marketing mix: Targeted promotions for focus communities in the United States. Nicotine Tob. Res..

[16] Hafez N., Ling P.M. (2006). Finding the Kool Mixx: How Brown & Williamson used music marketing to sell cigarettes. Tob. Control.

[17] Guo M., Ganz O., Cruse B., Navarro M., Wagner D., Tate B., Delahanty J., Benoza G. (2020). Keeping It Fresh With Hip-Hop Teens: Promising Targeting Strategies for Delivering Public Health Messages to Hard-to-Reach Audiences. Health Promot. Pract..

[18] Lee Y.O., Curry L.E., Fiacco L., Henes A., Farrelly M.C., Nonnemaker J.M., Hoffman L., Walker M.W. (2019). Peer crowd segmentation for targeting public education campaigns: Hip hop youth and tobacco use. Prev. Med. Rep..

[19] Moran M.B., Walker M.W., Alexander T.N., Jordan J.W., Wagner D.E. (2017). Why Peer Crowds Matter: Incorporating Youth Subcultures and Values in Health Education Campaigns. Am. J. Public Health.

[20] Lisha N.E., Jordan J.W., Ling P.M. (2016). Peer crowd affiliation as a segmentation tool for young adult tobacco use. Tob. Control.

[21] Moran M.B., Villanti A.C., Johnson A., Rath J. (2019). Patterns of Alcohol, Tobacco, and Substance Use among Young Adult Peer Crowds. Am. J. Prev. Med..

[22] Navarro M.A., Stalgaitis C.A., Walker M.W., Wagner D.E. (2019). Youth peer crowds and risk of cigarette use: The effects of dual peer crowd identification among hip hop youth. Addict Behav. Rep..

[23] Wagner D.E., Fernandez P., Jordan J.W., Saggese D.J. (2018). Freedom from Chew: Using Social Branding to Reduce Chewing Tobacco Use Among Country Peer Crowd Teens. Health Educ. Behav..

[24] Walker M.W., Navarro M.A., Hoffman L., Wagner D.E., Stalgaitis C.A., Jordan J.W. (2018). The Hip Hop peer crowd: An opportunity for intervention to reduce tobacco use among at-risk youth. Addict. Behav..

[25] Jordan J.W., Stalgaitis C.A., Charles J., Madden P.A., Radhakrishnan A.G., Saggese D. (2018). Peer Crowd Identification and Adolescent Health Behaviors: Results from a Statewide Representative Study. Health Educ. Behav..

[26] Harakeh Z., Bogt T. (2018). The Effect of Rap/Hip-Hop Music on Young Adult Smoking: An Experimental Study. Subst. Use Misuse.

[27] Ling P.M., Holmes L.M., Jordan J.W., Lisha N.E., Bibbins-Domingo K. (2017). Bars, Nightclubs, and Cancer Prevention: New Approaches to Reduce Young Adult Cigarette Smoking. Am. J. Prev. Med..

[28] Boslaugh S.E., Kreuter M.W., Nicholson R.A., Naleid K. (2005). Comparing demographic, health status and psychosocial strategies of audience segmentation to promote physical activity. Health Educ. Res..

[29] Guillory J., Henes A., Farrelly M.C., Fiacco L., Alam I., Curry L., Ganz O., Hoffman L., Delahanty J. (2020). Awareness of and receptivity to the fresh empire tobacco public education campaign among hip hop youth. J. Adolesc. Health.

[30] Nguyen N., Lisha N.E., Neilands T.B., Jordan J.W., Ling P.M. (2019). Differential Associations Between Anti-Tobacco Industry Attitudes and Intention to Quit Smoking Across Young Adult Peer Crowds. Am. J. Health Promot..

[31] Fallin A., Neilands T.B., Jordan J.W., Hong J.S., Ling P.M. (2015). Wreaking “havoc” on smoking: Social branding to reach young adult “partiers” in Oklahoma. Am. J. Prev. Med..

[32] Diffendal G. The Hard-To-Interview in the American Community Survey. Proceedings of the Annual Meeting of the American Statistical Association.

[33] Tourangeau R.E., Edwards B., Johnson T.P., Wolter K.M., Bates N. (2014). Hard-to-Survey Populations.

[34] Eaton D.K., Brener N.D., Kann L., Denniston M.M., McManus T., Kyle T.M., Roberts A.M., Flint K.H., Ross J.G. (2010). Comparison of paper-and-pencil versus Web administration of the Youth Risk Behavior Survey (YRBS): Risk behavior prevalence estimates. Eval. Rev..

[35] Chantala K., Kalsbeek W.D., Andraca E. (2005). Non-Response in Wave III of the Add Health Study.

[36] Substance Abuse and Mental Health Services Administration (2014). Getting Young Adult Survey Data: A Tale of Two States.

[37] Johnston L.D.M., Miech R.A., O’Malley P.M., Bachman J.G., Schulenberg J.E., Patrick M.E. (2019). Monitoring the Future National Survey Results on Drug Use, 1975–2018: Overview, Key Findings on Adolescent Drug Use.

[38] Ling P.M., Neilands T.B., Glantz S.A. (2009). Young adult smoking behavior: A national survey. Am. J. Prev. Med..

[39] Leventhal A.M., Strong D.R., Sussman S., Kirkpatrick M.G., Unger J.B., Barrington-Trimis J.L., Audrain-McGovern J. (2016). Psychiatric comorbidity in adolescent electronic and conventional cigarette use. J. Psychiatr. Res..

[40] Ling P.M., Lee Y.O., Hong J., Neilands T.B., Jordan J.W., Glantz S.A. (2014). Social branding to decrease smoking among young adults in bars. Am. J. Public Health.

[41] Kalkhoran S., Lisha N.E., Neilands T.B., Jordan J.W., Ling P.M. (2016). Evaluation of Bar and Nightclub Intervention to Decrease Young Adult Smoking in New Mexico. J. Adolesc. Health.

[42] Fallin A., Neilands T.B., Jordan J.W., Ling P.M. (2015). Social Branding to Decrease Lesbian, Gay, Bisexual, and Transgender Young Adult Smoking. Nicotine Tob. Res..

[43] Stalgaitis C.A., Navarro M.A., Wagner D.E., Walker M.W. (2020). Who Uses Tobacco Products? Using Peer Crowd Segmentation to Identify Youth at Risk for Cigarettes, Cigar Products, Hookah, and E-Cigarettes. Subst. Use Misuse.

[44] Lenk K., Rode P., Fabian L., Bernat D., Klein E., Forster J. (2012). Cigarette use among young adults: Comparisons between 2-year college students, 4-year college students, and those not in college. J. Am. Coll. Health.

[45] Moons K.G., Donders R.A., Stijnen T., Harrell F.E. (2006). Using the outcome for imputation of missing predictor values was preferred. J. Clin. Epidemiol..

[46] Jiang N., Ling P.M. (2013). Impact of alcohol use and bar attendance on smoking and quit attempts among young adult bar patrons. Am. J. Public Health.

[47] Hess C.A., Antin T.M., Annechino R., Hunt G. (2017). Perceptions of E-Cigarettes among Black Youth in California. Int. J. Environ. Res. Public Health.

[48] Hendlin Y.H., Veffer J.R., Lewis M.J., Ling P.M. (2017). Beyond the brotherhood: Skoal Bandits’ role in the evolution of marketing moist smokeless tobacco pouches. Tob. Induc. Dis..

[49] Kostygina G., Ling P.M. (2016). Tobacco industry use of flavourings to promote smokeless tobacco products. Tob. Control.

[50] Mejia A.B., Ling P.M. (2010). Tobacco industry consumer research on smokeless tobacco users and product development. Am. J. Public Health.

[51] Ling P.M., Glantz S.A. (2002). Why and how the tobacco industry sells cigarettes to young adults: Evidence from industry documents. Am. J. Public Health.

[52] Duke J.C., Lee Y.O., Kim A.E., Watson K.A., Arnold K.Y., Nonnemaker J.M., Porter L. (2014). Exposure to electronic cigarette television advertisements among youth and young adults. Pediatrics.

[53] Hendlin Y., Anderson S.J., Glantz S.A. (2010). Acceptable rebellion’: Marketing hipster aesthetics to sell Camel cigarettes in the US. Tob. Control.

[54] Hafez N., Ling P.M. (2005). How Philip Morris built Marlboro into a global brand for young adults: Implications for international tobacco control. Tob. Control.

[55] Pierce J.P., Sargent J.D., Portnoy D.B., White M., Noble M., Kealey S., Borek N., Carusi C., Choi K., Green V.R. (2018). Association Between Receptivity to Tobacco Advertising and Progression to Tobacco Use in Youth and Young Adults in the PATH Study. JAMA Pediatr..

[56] Knutzen K.E., Moran M.B., Soneji S. (2018). Combustible and Electronic Tobacco and Marijuana Products in Hip-Hop Music Videos, 2013–2017. JAMA Intern. Med..

[57] Ling P.M., Lisha N.E., Neilands T.B., Jordan J.W. (2020). Join the Commune: A Controlled Study of Social Branding Influencers to Decrease Smoking Among Young Adult Hipsters. Am. J. Health Promot..

[58] Slater M.D. (1996). Theory and method in health audience segmentation. J. Health Commun..

[59] Kreuter M.W., Wray R.J. (2003). Tailored and targeted health communication: Strategies for enhancing information relevance. Am. J. Health Behav..

[60] Jensen J.D., King A.J., Carcioppolo N., Krakow M., Samadder N.J., Morgan S. (2014). Comparing tailored and narrative worksite interventions at increasing colonoscopy adherence in adults 50–75: A randomized controlled trial. Soc. Sci. Med..

[61] Doll R., Peto R., Boreham J., Sutherland I. (2004). Mortality in relation to smoking: 50 years’ observations on male British doctors. BMJ.

[62] Sung H.Y., Wang Y., Yao T., Lightwood J., Max W. (2018). Polytobacco Use and Nicotine Dependence Symptoms Among US Adults, 2012–2014. Nicotine Tob. Res..

[63] Ali M., Gray T.R., Martinez D.J., Curry L.E., Horn K.A. (2016). Risk Profiles of Youth Single, Dual, and Poly Tobacco Users. Nicotine Tob Res.

